# Icariin alleviates osteoarthritis through PI3K/Akt/mTOR/ULK1 signaling pathway

**DOI:** 10.1186/s40001-022-00820-x

**Published:** 2022-10-17

**Authors:** Yan Chen, Xiaoli Pan, Jing Zhao, Chunyan Li, Yupei Lin, Yu Wang, Xu Liu, Mei Tian

**Affiliations:** 1 Department of Rheumatology and Immunology Department, Zunyi Medical University, Zunyi, 563006 China; 2 Department of Rheumatology and Immunology Department, Affiliated Hospital of Zunyi Medical University, 149 Dalian Road, Huichuan District, Zunyi, 563003 China; 3 Department of Rheumatology and Immunology Department, Peking University People’s Hospital, Beijing, 100044 China

**Keywords:** Icariin, Interleukin-1β, Autophagy, Chondrocytes, PI3K/Akt/mTOR/ULK1

## Abstract

**Objectives:**

This study aims to investigate the effects of Icariin (ICA) on interleukin-1β (IL-1β)-induced osteoarthritis (OA) and its potential mechanism of action.

**Methods:**

SW1353 chondrocytes were pretreated with ICA for 2 h, followed by stimulation with IL-1β to mimic OA. Expression levels of matrix metalloproteinases (MMP-3) and collagen II were determined using real-time PCR and Western blot assays. Autophagy activation (by ICA) or inhibition (by shRNA) was determined based on the expression levels of ULK1, Beclin-1, LC3-II/I, and p62, using Western blot analysis. The phosphorylation levels of PI3K, Akt, mTOR, and ULK1 were also detected using Western blot analysis.

**Results:**

IL-1β increased MMP-3 overproduction, induced collagen II degradation, and reduced the level of autophagy-associated proteins, including ULK1, Beclin-1, and LC3-II/I. In contrast, ICA pretreatment attenuated IL-1β-induced MMP-3 overproduction, increased collagen II expression, and induced expression of autophagy-related proteins. ICA also decreased PI3K, Akt, and mTOR phosphorylation, increased the production of ULK1, and induced autophagy. Short hairpin RNA-mediated knockdown of ULK1 led to activation of the PI3K/Akt/mTOR pathway, which reversed the protective effects of ICA.

**Conclusions:**

Our findings indicate that ICA can induce autophagy by regulating the PI3K/AKT/mTOR/ULK1 signaling pathway. This study suggests that ICA may be effective for treating OA.

## Introduction

Osteoarthritis (OA) is a chronic osteoarthropathy characterized by cartilage loss, subchondral bone remodeling, joint marginal osteophyte formation, and synovitis [1, 2]. With the aging population and lengthening survival rates, OA has become a common worldwide disease in the elderly population. The high incidence rate and lack of effective treatment of OA are the leading causes of disease burden [3, 4]. Currently, there are more than 300 million OA patients worldwide. The life quality of OA patients is seriously impaired due to severe limitations in limb function [5]. Recent research has revealed that obesity, aging, strenuous exercise, inflammation, previous joint trauma, metabolic factors, and genetic susceptibility are contributing factors in the pathogenesis of OA [6, 7].

Recent studies revealed that the underlying mechanisms of OA are associated with autophagy, which is crucial for maintaining cartilage integrity through the clearance of misfolded proteins, damaged organelles, or dysfunctional cell components [8]. Increasing evidence has also indicated that deficiency in the process of autophagy is significantly involved in the pathogenesis of OA [9]. Moreover, defects in autophagy regulation in chondrocytes and aging cartilage have been observed in OA models [10]. In the early stage of OA, autophagy can actively protect chondrocytes [11], while in the later stage, autophagy can be active together with apoptosis as an alternative mechanism of cell death that can induce the aging process [12]. Therefore, autophagy could be a target for OA research.

The pathophysiological mechanisms of OA are very complex and, as such, are not entirely understood. Thus, there is no specific drug that effectively treats OA. Natural products with therapeutic and preventive effects on bone resorption have recently attracted increasing attention, because they may be more suitable for long-term use than traditional therapeutic compounds [13, 14]. For example, Epimedi, a main active component of Icariin (ICA), is a flavonoid used to treat bone and joint diseases for many centuries in China [15]. However, the exact mechanism of action of ICA is not completely clear. In a mouse model of OA, ICA can reduce cartilage destruction, promote chondrocyte differentiation, and upregulate the expression of parathyroid hormone-related proteins [16]. Mi et al. [17] reported that ICA reduced nuclear factor-κB (NF-κB) signal-mediated chondrocyte apoptosis through activating autophagy.

To better understand the pharmacological effects of ICA, we investigated the effects and underlying mechanism of ICA using an interleukin-1β (IL-1β)-induced OA in vitro model.

## Materials and methods

### Chemical reagents and antibodies

Dulbecco's modified Eagle’s medium (DMEM), fetal bovine serum (FBS), and 0.25% trypsin solution were purchased from Gibco (Grand Island, NY, USA). ICA and penicillin–streptomycin mixture were obtained from Solarbio Science and Technology Co., Ltd. (Beijing, China). Antibodies targeting LC3B, Beclin 1, p62/SQSTM1, and ULK1, as well as phosphorylated (p-) antibodies, including p-PI3K, p-AKT, and p-mTOR, were purchased from the American CST company. Antibodies targeting PI3K, AKT, mTOR, matrix metalloproteinases 3 (MMP3), collagen II, and GAPDH were purchased from Hua’an Science and Technology Co., Ltd. (Hangzhou, China). Recombinant human IL-1β was a product of the Peprotech Company (Cranbury, NJ, USA).

### Cell culture

The SW1353 cell line was derived from humerus chondrosarcoma, which is considered an appropriate in vitro cell model to investigate the function of human primary chondrocytes. This cell line was obtained from the Chinese Academy of Sciences and cultured in DMEM supplemented with 10% FBS, under 5% CO_2_ at 37 ℃, with 0.5% penicillin/streptomycin and 1% glutamine. In addition, SW1353 cells were stimulated with IL-1β to establish OA in vitro.

### Cell viability assays

Cell viability was determined using the CCK8 assay. Briefly, SW1353 cells (5 × 10^3^ cells/well) were plated onto a 96-well plate overnight. After the cells adhered, pretreatment with ICA was performed at different concentrations (0, 5, 10, 20, 40, 80, and 100 μM) for 2 h, followed by incubation with IL-1β at different concentrations (0, 5, 10, and 20 ng/mL) at 37 ℃ for 24 h [18]. Then, 10 μL of the CCK-8 reagent was added to each well, and the cells were incubated for another 2 h. Finally, absorbance at 450 nm was measured using a microplate reader, and the measurements were repeated three times to calculate the average value for cell viability.

### Western blot analysis

Protein lysates were prepared from cultured SW1353 cells using a protein extraction kit, and the concentrations of sample lysates were determined using the BCA assay. After completion of SDS–PAGE electrophoresis, separated proteins in the gel were transferred onto a PVDF membrane. After blocking with 5% non-fat milk for 2 h at room temperature, the membranes were incubated overnight at 4℃ with the following primary antibodies: collagen II, MMP-3, LC3, p62, Beclin1, ULK1, PI3K, AKT, mTOR, p-PI3K, p-AKT, p-mTOR, and GAPDH (as the internal control). On the next day, the membranes were incubated with horseradish peroxidase-conjugated secondary antibodies (according to the animal species of the primary antibodies) for 1 h at room temperature with shaking. The protein bands were detected with the ECL Western blot reagent. The blots were visualized using the Davinch-Chemi™ imaging system. Quantity One 1-D analysis software v4.52 (BioRad, Philadelphia, USA) was used to quantify the relative optical intensity of the protein bands.

### RNA isolation and real-time PCR

Total RNA from SW1353 cells was isolated using a total RNA extraction kit. The RNA was reverse transcribed with the PrimeScript RT reagent kit (Promega Corporation). Real-time PCR was performed to measure mRNA levels in the samples using the SYBR Green master mix on the CFX Connect System (Bio-Rad Laboratories, Inc., Hercules, CA, USA). The primer sequences used in this study are shown in Table 1. The average *Ct* values calculated from triplicate PCRs were normalized to the average *Ct* values of GAPDH. These normalized values were then used to calculate a gene expression value using the formula 2^−(meanΔΔCt)^.

**Table 1:** List of the primers used in quantitative PCR

Gene	Forward primer	Reverse primer
ULK1	5′-GCATTAACAAGAAGAACCTCGCCAAG-3′	5′-GCATTAACAAGAAGAACCTCGCCAAG-3′
MMP-3	5′-AAGACAGCAAGGCATAGAGACAACATAG -3′	5′-ACAGCAACAGTAGGATTGGAAGACTC-3′
Beclin1	5′-ACATCTGGCACAGTGGACAGTTTG -3′	5′-AGCATGGAGCAGCAACACAGTC-3′
Collagen-II	5′-GAGGGCAACAGCAGGTTCAC-3′	5′-GCCCTATGTCCACACCAAATTC-3′
Aggrecan	5′-TGGCATTGAGGACAGCGAAG-3′	5′-TCCAGTGTGTAGCGTGTGGAAATAG-3′
GAPDH	5′-CAAGTTCAACGGCACAGTCAAG-3′	5′-ACATACTCAGCACCAGCATCAC-3′

### Lentiviral shRNA infection

The lentiviral particles of either ULK1-targeted shRNA or control shRNA were obtained from Hanbio Tech (Shanghai, China). Viral infection of SW1353 cells was performed with a multiplicity of infection (MOI) of 50, according to the manufacturer’s instructions. At 72 h after transduction, cells transfected with shRNA were selected with puromycin for an additional 48 h. Finally, the surviving cells in which ULK1 shRNA was stably expressed (confirmed by Western blot analysis) were selected for further experiments.

### Statistical analysis

SPSS 20.0 statistics software was used for the analysis of all data. Data were obtained from at least three separate experiments and are expressed as mean ± standard deviation (SD). One-way analysis of variance for multiple groups or Student’s *t* test for two groups was used for data analysis with comparisons. A *P* < 0.05 was considered statistically significant.

## Results

### ICA rescues IL-1β-mediated growth inhibition in SW1353 cells

**Fig. 1:**
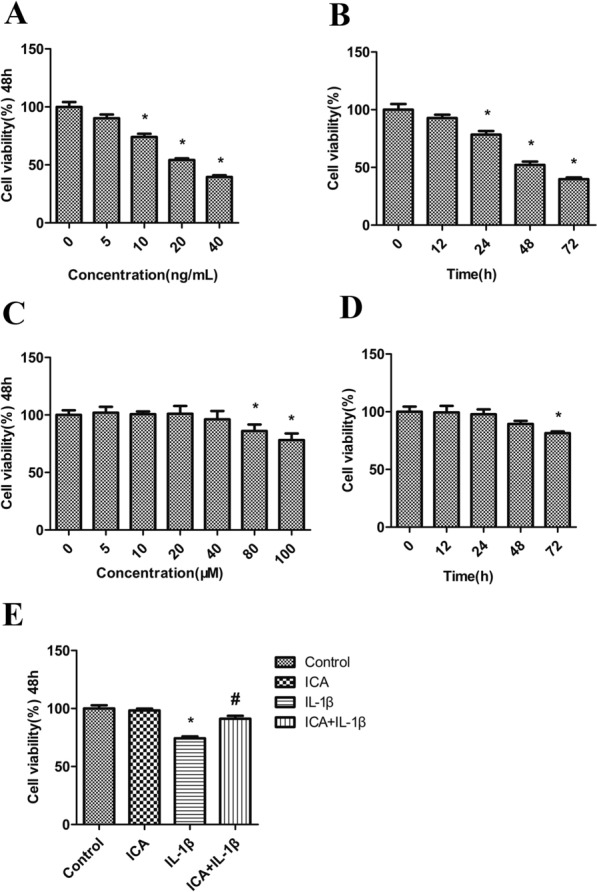
ICA rescues IL-1β-mediated growth inhibition in SW1353 cells. **A** Dose-dependent effect of IL-1β on cell viability of SW1353 cells (48 h). **B** Time-dependent effect of IL-1β (20 ng/mL) on cell viability of SW1353 cells (12, 24, 48, and 72 h). **C** Dose-dependent effect of ICA on cell viability of SW1353 cells (48 h). **D** Time-dependent effect of ICA (40 μM) on cell viability of SW1353 cells (12, 24, 48, and 72 h). **E** Impact of ICA pretreatment on cell viability of IL-1β-treated SW1353 cells (48 h)

The effect of IL-1β on cell viability in the cultured chondrocytes was determined using the CCK8 assay. SW1353 cells were treated with IL-1β at different concentrations (0, 5, 10, 20, and 40 ng/mL) for 48 h (Fig. 1A). IL-1β treatment significantly decreased cell viability at the higher concentrations (10, 20, and 40 ng/mL). Compared to the vehicle control (100%), the survival rates of IL-1β-treated SW1353 cells were 74.0%, 54.2%, and 39.5%, at 10, 20, and 40 ng/mL, respectively. IL-1β (20 ng/mL) treatment for 12 h did not affect cell viability. However, its inhibitory effect could be detected at 24, 48, and 72 h (Fig. 1B). Considering that 48 h treatment of IL-1β (20 ng/mL) significantly decreased cell viability by approximately 50%, we selected 20 ng/mL for 48 h for all further experiments.

To estimate the effect of ICA on the cell viability of chondrocytes, SW1353 cells were treated with various concentrations of ICA (0, 5, 10, 20, 40, 80, and 100 μM) for 48 h (Fig. 1C). The lower doses of ICA (0–40 μM) had no significant effect on cell proliferation, whereas the higher doses of ICA (80–100 μM) significantly inhibited cell growth compared to the untreated group. Moreover, at 40 μM, ICA had no effect on cell viability after 12, 24, and 48 h of treatment compared to the control group, except for 72 h treatment. Therefore, the optimal condition for further experiments was ICA (40 μM) treatment for 48 h (Fig. 1C, D).

To evaluate the influence of ICA treatment on the growth inhibition of SW1353 cells caused by IL-1β, cells were pretreated with ICA (40 μM for 2 h) followed by IL-1β (20 ng/mL) incubation for 48 h (Fig. 1E) as described previously. Cell viability following IL-1β treatment was markedly decreased compared to the control. However, ICA pretreatment increased cell viability. These results demonstrate that pretreatment with ICA can significantly rescue IL-1β-mediated growth inhibition in SW1353 cells.

### ICA inhibits IL-1β-induced MMP-3 activation and promotes collagen II expression

**Fig. 2:**
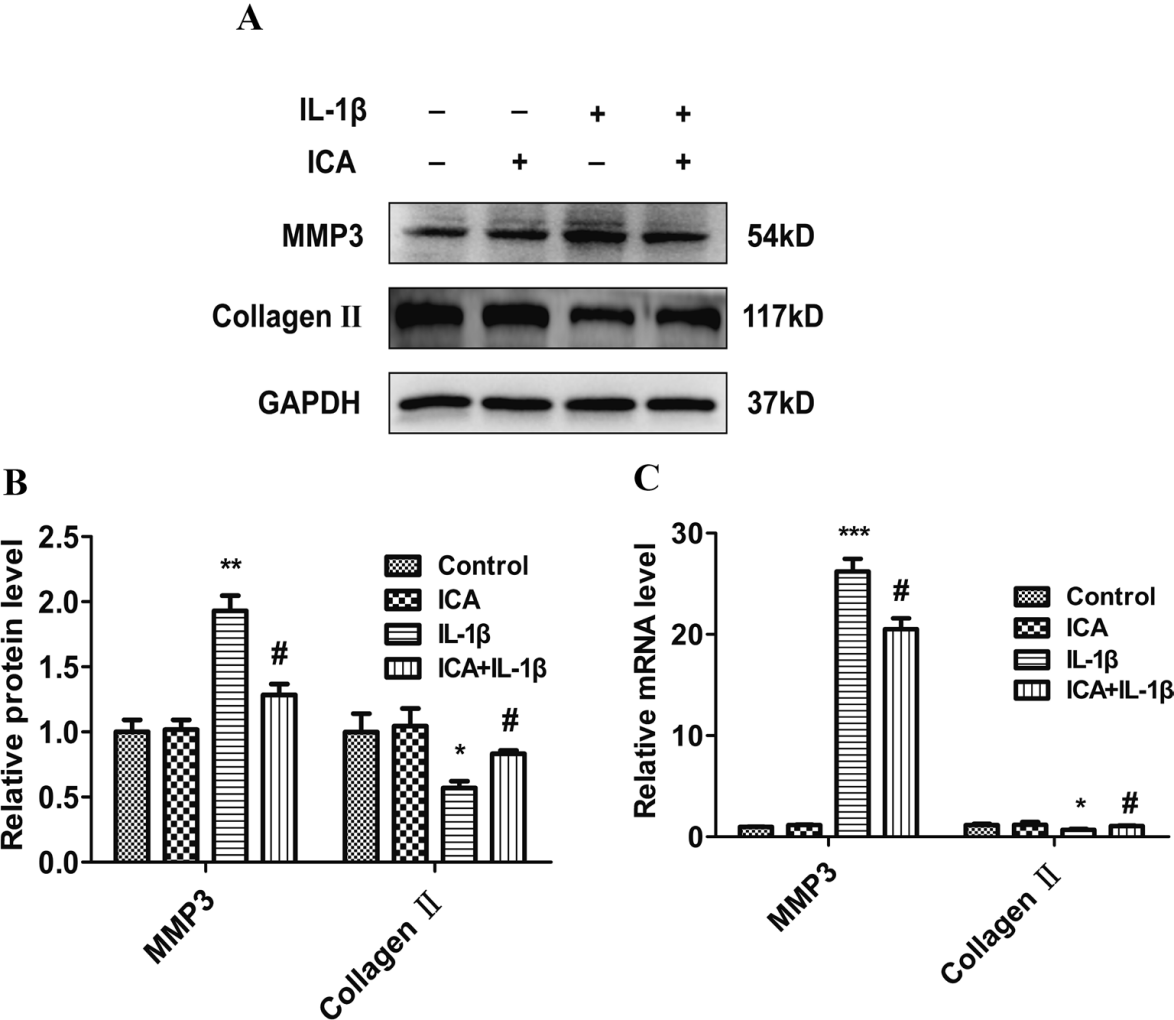
Effects of ICA on IL-1β-induced MMP-3 and collagen II expression. **A** Representative Western blot images of MMP-3 and collagen II. **B** Quantitation of MMP-3 and collagen II. **C** MMP-3 and collagen II mRNA levels determined by real-time PCR. Data are presented as means ± SD (*n* = 3). *P < 0.05 versus control group, *# P* < 0.05 versus IL-1β group

The effect of ICA on IL-1β-induced MMP-3 production and the expression of collagen II was evaluated using Western blot and real-time PCR assays. MMP-3 protein production was significantly elevated in IL-1β-treated cells compared to the control cells (*P* < 0.01), whereas the expression of collagen II was significantly reduced in IL-1β-treated cells (*P* < 0.05). However, pretreatment with ICA markedly inhibited IL-1β-induced MMP-3 overproduction and promoted collagen II expressions (*P* < 0.05) (Fig. 2A, B). Furthermore, PCR results showed that IL-1β treatment decreased collagen II mRNA levels, while ICA promoted collagen II expressions (*P* < 0.05) (Fig. 2C). The results for MMP-3 expression were consistent between Western blot and real-time PCR analysis results. Our results demonstrate that ICA can alleviate IL-1β-induced inflammation, thereby promoting collagen formation.

### ICA induces autophagy in OA chondrocytes

**Fig. 3:**
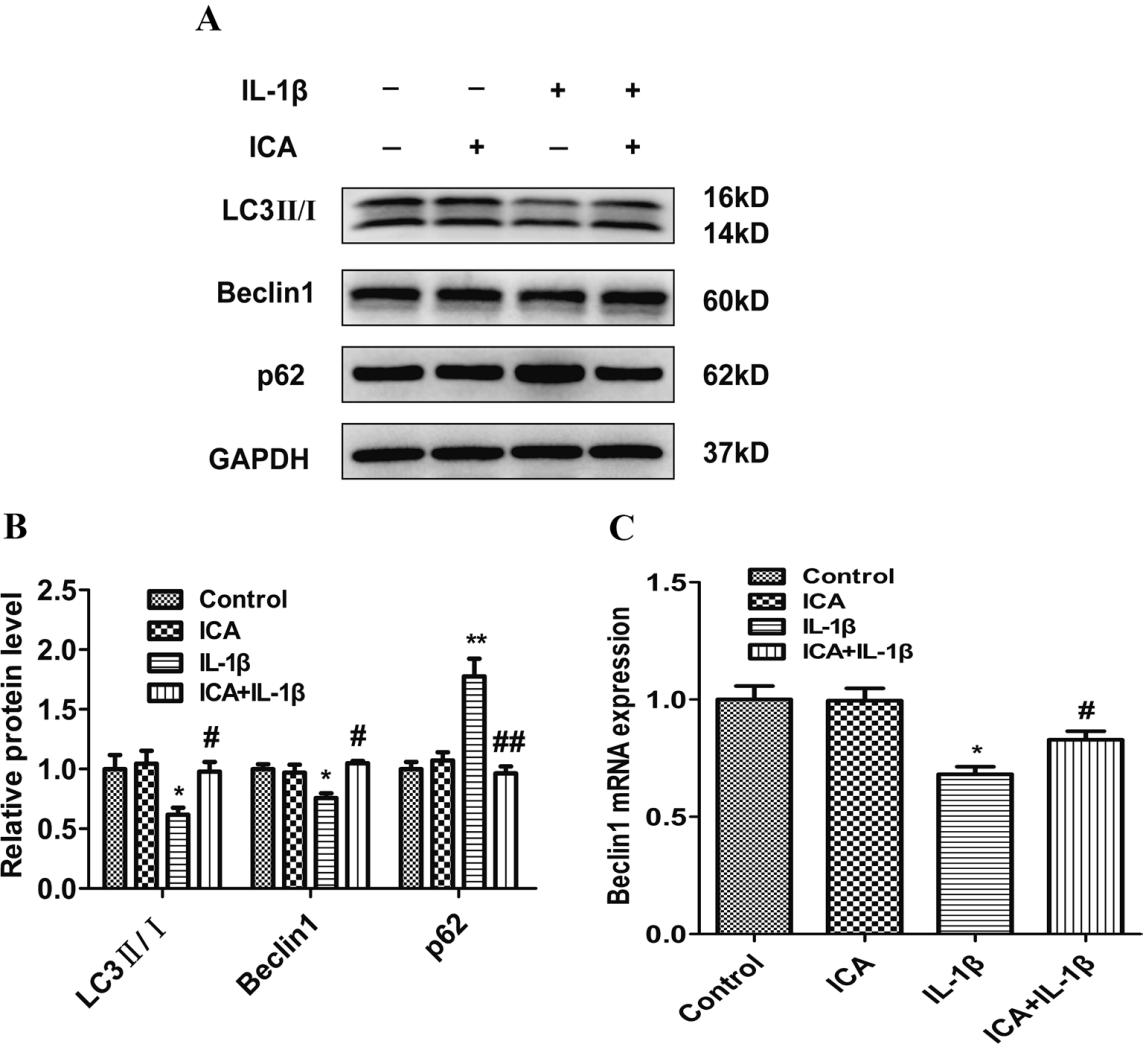
ICA induces autophagy in OA chondrocytes. **A** Representative Western blot images of LC3-II/I, Beclin 1, and p62. **B** Quantitation of LC3-II/I, Beclin 1, and p62. **C** Beclin 1 mRNA level was determined using real-time PCR. Data are presented as means ± SD (*n* = 3). *P < 0.05 versus control group, #*P* < 0.05 versus IL-1β group

Autophagy is a critical biological process for the self-repairing function of cells. To this end, we evaluated the effect of ICA on autophagy activation in OA chondrocytes. Relative to the control, protein expression of LC3 II/I and Beclin 1 was significantly decreased, while p62 was significantly increased in the IL-1β treatment group (Fig. 3A, B). Moreover, protein expression of LC3 II/I and Beclin 1 was significantly upregulated in the ICA groups compared to the IL-1β group (*P* < 0.05). However, p62 protein expression was significantly downregulated in the ICA groups compared to the IL-1β group (*P* < 0.01) (Fig. 3A, B). PCR results revealed that IL-1β downregulated Beclin1 levels, whereas ICA upregulated Beclin 1 (*P* < 0.05) (Fig. 3C). These results indicate that ICA strongly induces autophagy in SW1353 cells.

### Knockdown of ULK1 affects the anti-inflammation effects of ICA in OA chondrocytes

**Fig. 4:**
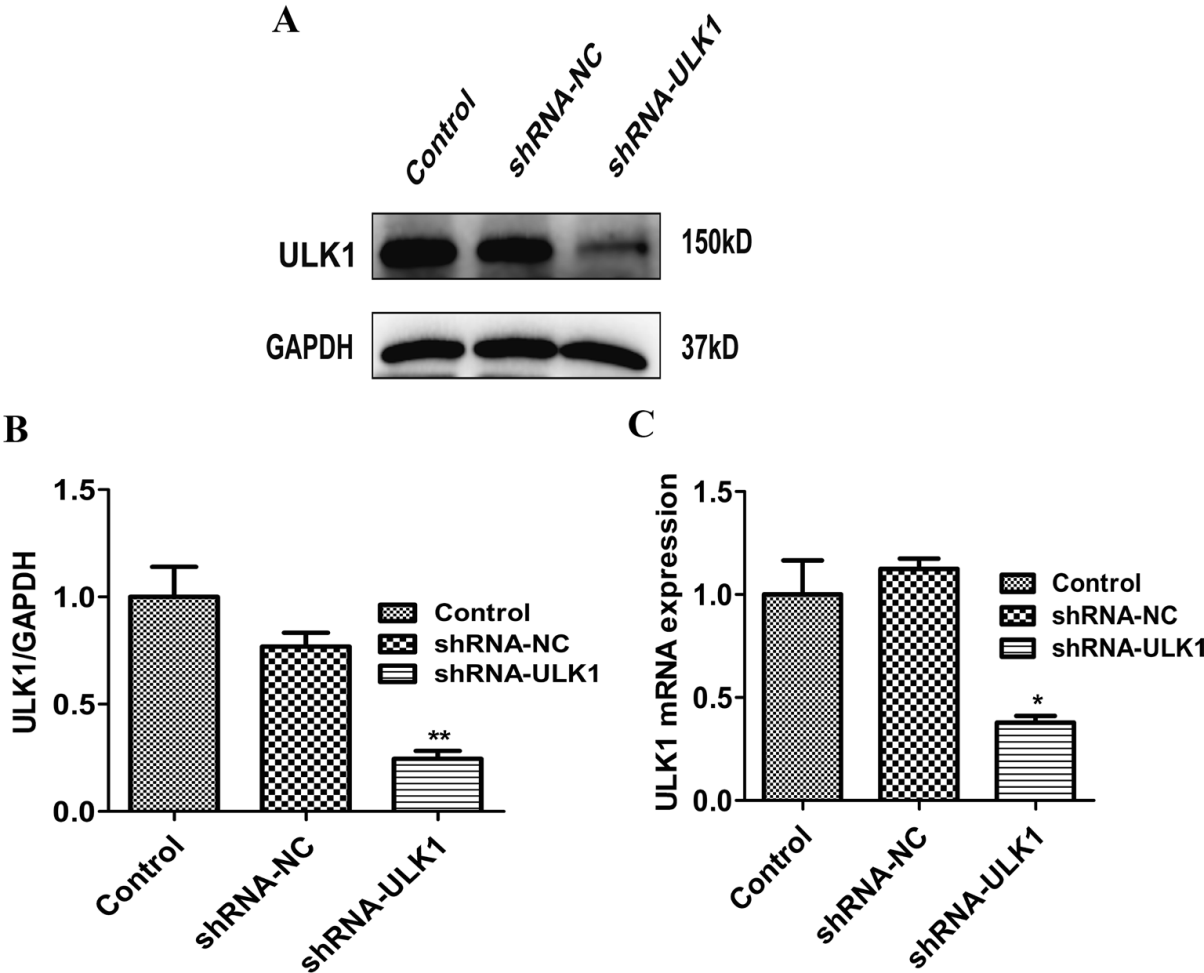
Effect of shRNA/ULK1 on ULK1 expression. **A** Representative Western blot image of ULK1. **B** Quantitation of ULK1. **C** ULK1 mRNA levels were determined using real-time PCR. Data are presented as means ± SD (*n* = 3). * *P* < 0.05 versus control group

We next investigated the mechanism underlying the anti-inflammation effects of ICA via the regulation of autophagy. Because ULK1 is a pivotal autophagy protein, we knocked down ULK1 expression using shRNA and investigated if ULK1 contributes to the anti-inflammation effects of ICA in OA chondrocytes. ULK1 protein expression and mRNA levels were measured using Western blot and real-time PCR assays. There was a statistically significant reduction in ULK1 protein expression and mRNA levels in the shRNA/ULK1 group compared to the shRNA/NC group and non-transfection control group (*P* < 0.05) (Fig. 4A–C), indicating that ULK1 expression in SW1353 cells was successfully knocked down.

**Fig. 5:**
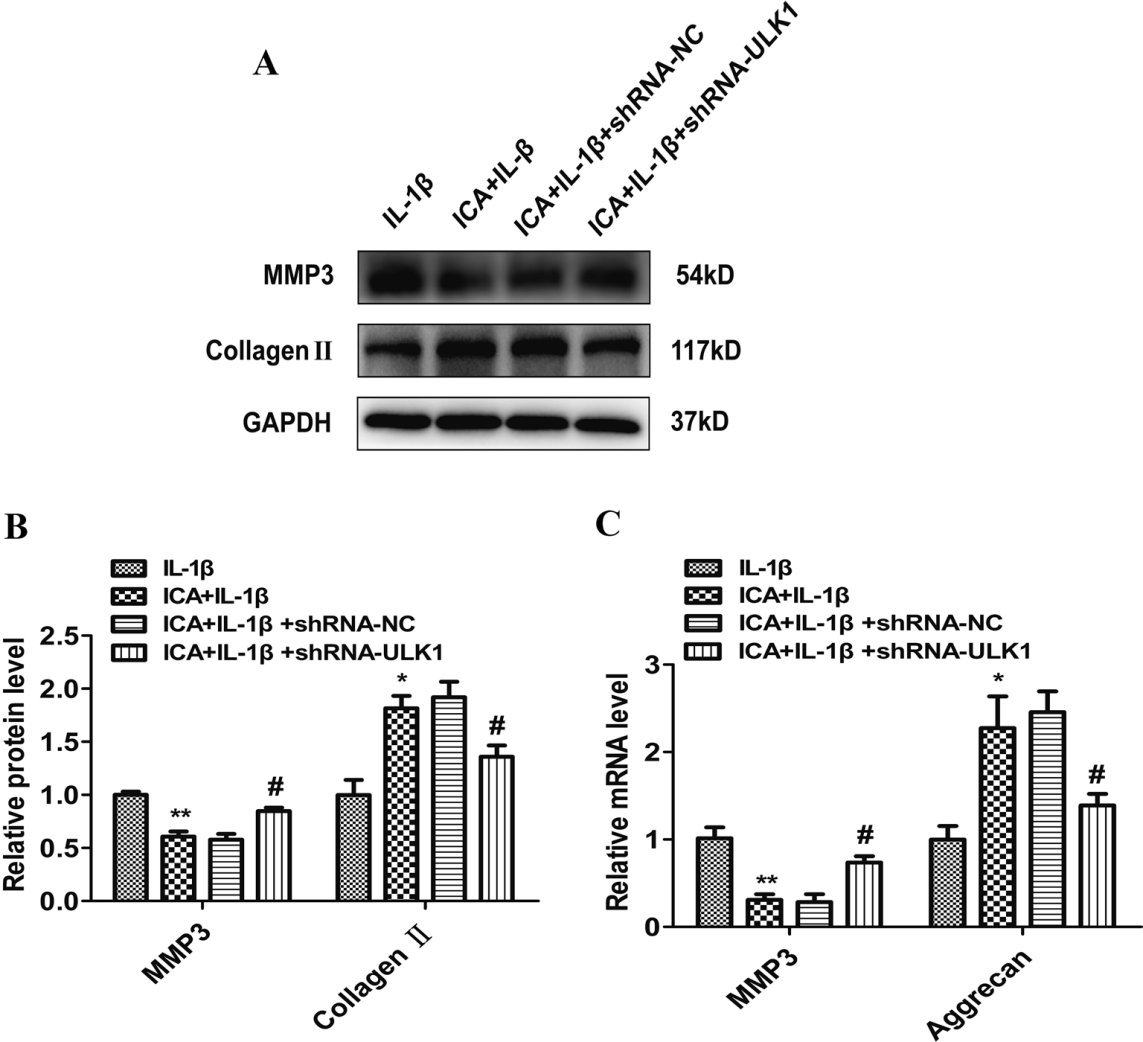
ULK1 knockdown promotes IL-1β-induced inflammation in OA chondrocytes. **A** Representative Western blot images of MMP-3 and collagen II after ULK1 knockdown. **B** Quantitation of MMP-3, collagen II. **C** MMP-3, aggrecan mRNA levels were determined using real-time PCR. Data are presented as means ± SD (*n* = 3). *P < 0.05 versus control group (IL-1β group), #*P* < 0.05 versus shRNA/NC group

Next, we investigated the effects of ULK1 knockdown on ICA-induced inflammatory markers. We found that MMP-3 levels were decreased, but collagen II levels were increased in the ICA group (with IL-1β incubation) compared to the IL-1β (alone) group (*P* < 0.05) (Fig. 5A–C). In the shRNA/ULK1 group, there was a significant increase in MMP-3 but a decrease in collagen II compared to the shRNA/NC group (*P* < 0.05) (Fig. 5A, B). PCR analysis also showed that there was a decrease in MMP-3 and an increase in aggrecan in the ICA group compared to the control group (IL-1β alone) (*P* < 0.05) (Fig. 5C). However, different from the results of ICA treatment in OA chondrocytes, there was a significant increase in MMP-3 and a decrease in aggrecan after ULK1 knockdown compared to the shRNA/NC group (*P* < 0.05) (Fig. 5B, C). These results illustrate that ICA can alleviate the inflammatory response induced by IL-1β. However, after ULK1 knockdown, ICA-induced autophagy in OA chondrocytes was attenuated. As a result, the anti-inflammation effects of ICA in OA chondrocytes were abolished, implying that ULK1 is involved in ICA-mediated autophagy and contributes to the anti-inflammation effects of ICA in OA chondrocytes.

### ULK1 knockdown attenuates autophagy in OA chondrocytes

**Fig. 6:**
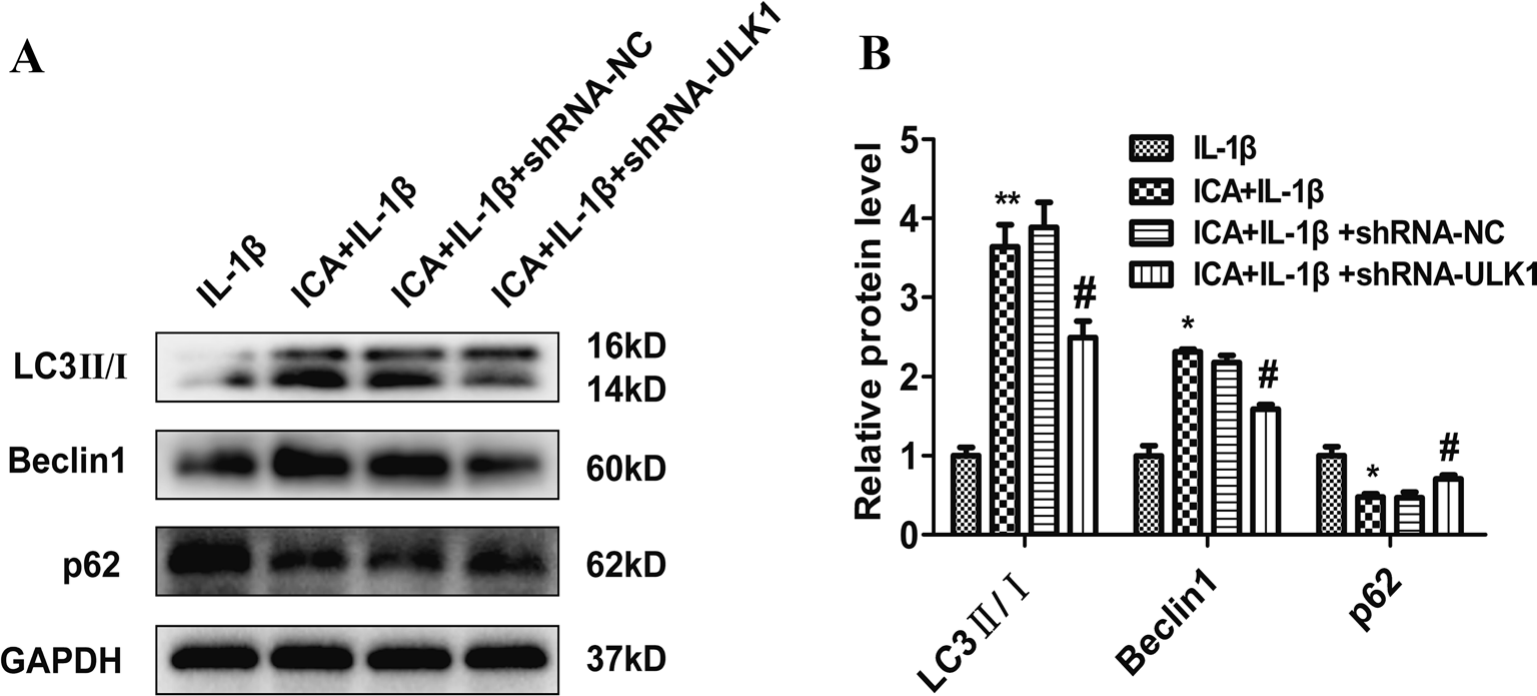
ULK1 knockdown attenuates autophagy in OA chondrocytes. **A** Representative Western blot images of Beclin 1, LC3-II/I, and p62 after ULK1 knockdown. **B** Quantitation of p62, Beclin 1, LC3-II/I. Data are presented as means ± SD (*n* = 3). *P < 0.05 versus control group (IL-1β group), #*P* < 0.05 versus shRNA/NC group

To elucidate the role of ICA in the activation of autophagy, the expression levels of autophagy-associated proteins, including Beclin 1, LC3-II/I, and p62 in OA chondrocytes, were determined using immunoblot analysis. There was an increase in Beclin 1 and LC3-II/I in the ICA group (also with IL-1β) compared to the IL-1β (alone) groups (*P* < 0.05). The increase in Beclin 1 and LC3-II/I was accompanied by a decrease in p62 (*P* < 0.05) (Fig. 6A, B), providing evidence of autophagy activation. In contrast, knockdown of ULK1 led to a significant decrease in protein expression of Beclin 1 and LC3-II/I, as well as a significant increase in p62 compared to the shRNA/NC group (*P* < 0.05) (Fig. 6A, B). These results illustrate the involvement of both ICA and ULK1 in the regulation of autophagy, since ULK1 knockdown attenuated ICA-induced autophagy in OA chondrocytes.

### ULK1 knockdown activates the PI3K/AKT/mTOR signaling pathway

**Fig. 7:**
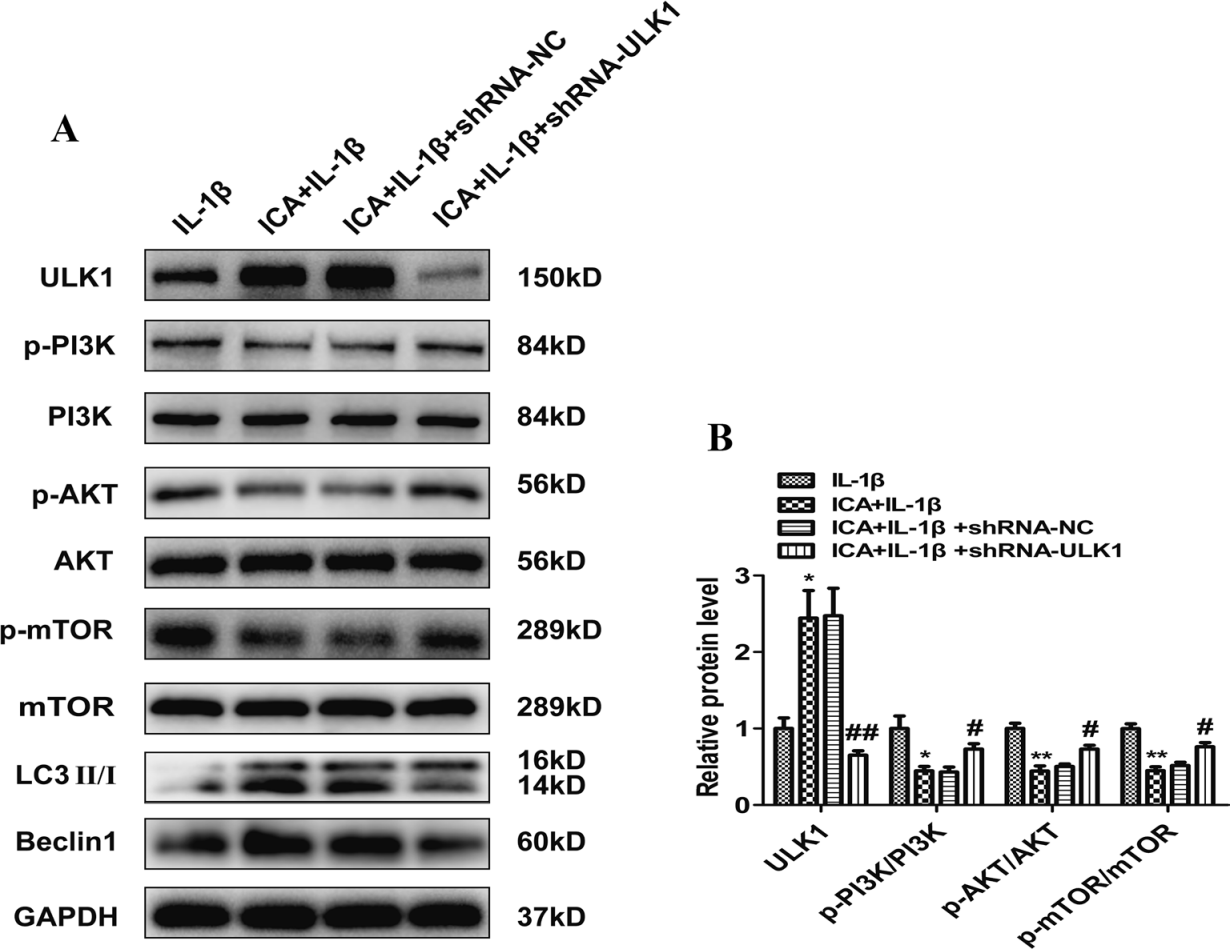
ULK1 knockdown activates the PI3K/AKT/mTOR signaling pathway. **A** Representative Western blot images of ULK1, p-PI3K, PI3K, p-AKT, AKT, p-mTOR, and mTOR. **B** Quantitation of ULK1, p-PI3K/PI3K, p-AKT/AKT, p-mTOR/mTOR. Data are presented as means ± SD (*n* = 3). *P < 0.05 versus control group (IL-1β group), #*P* < 0.05 versus shRNA/NC group

To determine if the protective effect of ICA on OA chondrocytes is mediated through regulation of the PI3K/Akt/mTOR signaling pathway, we measured phosphorylation of PI3K, AKT, and mTOR using immunoblot assays. The ICA group demonstrated an increase in ULK1, LC3, and Beclin1, as well as a decrease in the ratio of phosphorylated (an index of activation) to the total expression of PI3K, AKT, and mTOR compared to the ratios in the IL-1β group (*P* < 0.05) (Fig. 7A, B). However, there were no statistically significant differences in the expression levels of PI3K, Akt, and mTOR in each group. These results suggest that ICA can promote autophagy by inhibiting the activation of the PI3K/AKT/mTOR signaling pathway. ULK1 protein expression was also lower in the shRNA/ULK1 group compared to the shRNA/NC group (*P* < 0.05) (Fig. 7A, B). However, compared to the IL-1β + shRNA/NC group, the ICA + IL-1β + shRNA/ULK1 group showed a significant increase in the levels of p-PI3K, p-AKT1, and p-mTOR and a drastic decrease in the levels of both LC3-II/I and Beclin 1. These results indicate that both the inhibitory effect of ICA on the PI3K/AKT/mTOR signaling pathway and its activation effect on autophagy were abolished after ULK1 knockdown, implying that ULK1 is an essential player in the ICA-mediated anti-inflammation effect on OA chondrocytes.

## Discussion

As the elderly population increases, the incidence rate of OA is also increasing, which has attracted global attention. At present, there is no specific drug to treat OA effectively. Current therapies for OA, such as intra-articular injection of sodium hyaluronate, can only partially relieve pain and certain trauma. Over-the-counter medications are available, but NSAIDs have side effects of the cardiovascular, renal, digestive tract, and intestinal microbiota disorders [19]. Opioid analgesics are problematic because of their addictive properties and cannot be widely used. Flavonoids can improve antioxidation and scavenge free radicals and have been shown to reduce the speed of aging. The flavonoid ICA has a variety of biological functions, such as anti-oxidation [20, 21], anti-tumor, cardiovascular protection, anti-inflammatory [22], anti-microbial, immune enhancement, anti-aging, and estrogen-like effects [23]. Thus, flavonoids can be used to treat OA, as it is an aging-related degenerative disease.

Although cartilage deterioration is the most significant factor in OA, the synovium, articular ligament, and subchondral bone can also contribute to OA. Synovitis and systemic inflammation play an essential role in the occurrence and development of OA [24]. Previous studies demonstrated that both inflammation and extracellular matrix (ECM) degradation are 2 crucial factors in OA development. The most important inflammatory mediators in the pathogenesis of OA are IL-1β, TNF-α, and IL-6. In response to cytokine stimulation, inflammatory cells are attracted to the involved joints, where there are more secreted inflammatory factors leading to disease acceleration [25]. Among the cytokines related to inflammation, IL-1β promotes the generation and release of several inflammatory mediators, including TNF-α, inducible nitric oxide synthase (iNOS), and MMPs, all of which contribute to chondrocyte dysfunction and ECM degradation [26–28]. For this reason, IL-1β is widely applied to establish models of cellular OA [29, 30]. Treatment with IL-1β in SW1353 cells is not an ideal cell model, but it is a good tool for investigating inflammation. This study focused on both inflammation and autophagy and, therefore, chose this model. The occurrence and development of OA are related to the degeneration of cartilage structure and changes in the ECM. MMP-3 and type II collagen are recognized as biomarkers of OA [31]. The severity of OA is related to the accumulation of MMP3 and the decrease of type II collagen. Our study confirmed excessive MMP-3 production and collagen II degradation in the IL-1β-induced OA model. Using this model, we found that ICA can reduce IL-1β-dependent MMP-3 overproduction and collagen II degradation. The results also showed that ICA could promote the proliferation of chondrocytes, thereby affecting the ECM environment of chondrocytes and antagonizing IL-1β-induced chondrocyte degeneration (Fig. 2).

Because of the increased secretion of a large number of MMPs and platelet reactive proteins, autophagy in chondrocytes helps remove harmful substances and maintain homeostasis, prolonging the survival of chondrocytes and alleviating symptoms of OA. However, chondrocytes cannot maintain the function of autophagy in the development of OA [12, 32]. For example, ULK1, a serine/threonine kinase, is an essential autophagy-related gene in human cells. The expression of ULK1 can be directly regulated by the autophagy central regulatory molecule mTOR [33]. Under stress conditions, the inactivation of mTOR can promote the formation of the ULK complex, and the ULK1/FIP200/Atg13 complex is essential for the formation of autophagosomes [33, 34]. In addition, ULK1, Beclin1, and LC3 are commonly used as biomarkers for autophagy processes [35]. Both human knee cartilage degeneration and aging in mice are associated with decreased expression of autophagy-related proteins, such as LC3, Beclin 1, and ULK1 [36]. Our study showed that ICA could attenuate IL-1β-mediated growth inhibition of chondrocytes, as well as the excessive production of MMP-3 and degradation of collagen II. We also found an increase in the expression of autophagy proteins, including LC3 and Beclin1, while p62 was significantly decreased, all of which may be associated with the anti-inflammation effects of ICA in OA chondrocytes (Fig. 3). To verify that ICA induces autophagy in chondrocytes, we knocked down the ULK1 gene in our in vitro model. Research shows that when ULK1 is knocked out by specific lentiviral shRNA, IL-1β may promote the excessive accumulation of MMP-3 and reduce collagen II degradation even after ICA pretreatment in an OA cell model, suggesting that ICA may lose its protective effect in OA chondrocytes in the absence of ULK1 (Fig. 5).

The regulatory mechanisms of autophagy are very complex. The PI3K/AKT/mTOR pathway, an essential intracellular signaling pathway, is closely related to chondrocyte apoptosis and autophagy [37]. Moreover, activation of the PI3K/Akt/mTOR signaling pathway is also critical in the occurrence and development of OA [38]. Huang et al. [39] found that NOV/CCN3, a multi-functional protein, can promote autophagy of rat articular chondrocytes, reduce inflammation, and improve IL-1β-induced catabolism by suppressing activation of the PI3K/Akt/mTOR signaling pathway. It has been reported that four-octyl itaconate enhanced chondrocyte autophagy and improved OA by inhibiting the PI3K/AKT/mTOR signaling pathway [40]. Our study also demonstrated that ICA could inhibit IL-1β-induced phosphorylation of PI3K, Akt, and mTOR, attenuate the excessive production of MMP-3, and promote the production of collagen II. ICA treatment also increased the expression of ULK1. Here, we showed that ULK1 knockdown by a specific lentiviral shRNA in OA chondrocytes increased phosphorylation of PI3K/Akt/mTOR, indicating that the PI3K/Akt/mTOR signaling pathway is activated and that ICA loses its protective effect in OA chondrocytes in the absence of ULK1 (Fig. 7).

Rapamycin is a pharmacological inhibitor of mTOR. Recent studies revealed that rapamycin partially inhibits mTOR through allosteric inhibition of the mTOR complex 1 (mTORC1) but not mTOR complex-2 (mTORC2). Rapamycin is also an activator of autophagy. For example, Everolimus is an allosteric inhibitor of mTORC1, widely known for its potent autophagy-stimulating properties. Takayama et al. [41] found that local intra-articular injection of rapamycin in a mouse OA model can reduce the expression of mTOR and MMP-13 and activate LC3, suggesting that rapamycin could be used for the prevention of OA. However, this drug has many side effects, such as diarrhea, weight loss, proteinuria, anemia, allergy, hypercholesterolemia, and hypertriglyceridemia [36], which might limit its clinical application.

Our findings are consistent with other reports. For instance, the use of rapamycin and the autophagy inhibitor (3-methyladenine) confirmed that ICA could improve the course of OA by regulating the PI3K/Akt/mTOR signaling pathway [42]. The unique aspect of our study is that we used shRNA-mediated ULK1 knockdown to confirm that the anti-inflammation effects of ICA on OA are associated with autophagy and activation of the PI3K/Akt/mTOR signaling pathway. On the other hand, the Xianling Gubao capsule (XLGBC) is a traditional medicine for the treatment of postmenopausal osteoporosis [43]. The main component of XLGBC is epimedium, which has good efficacy and safety [44]. This previous study provided more favorable evidence to further explore the use of XLGBC in future OA treatment.

Of course, our study has certain limitations. First, selecting IL-1β-induced SW1353 cells to mimic OA is an excellent inflammatory model; however, this is not a good OA chondrocyte model. Second, due to the limitation of the experimental design, autophagy agonists, such as rapamycin, were not used as positive controls. In addition, there were only in vitro experiments in this study, which lacked in vivo animal and translational studies in the clinic due to time limitations. Despite the apparent limitations of a cell model to study a joint disease that has also been described as a systemic disease, we still obtained preliminary results using IL-1β-treated SW1353 chondrocytes to mimic the inflammatory environment found in OA. Our findings could help design further in vivo animal and translational studies.

## Conclusions

Our study demonstrates that the ICA can alleviate IL-1β-induced OA by regulating the PI3K/Akt/mTOR/ULK1 signaling pathway. These findings indicate that ICA may be a potent and effective therapeutic strategy for treating OA.

## Data Availability

The data sets are not publicly available due to restrictions used under the license for the current study. However, there are available on reasonable request from the corresponding author.

## Abbreviations

ATG: Autophagy-related gene;
AKT: Protein kinase B
Beclin-1: Becn1 gene
CCK8: Cell counting kit-8
DMEM: Dulbecco’s modified eagle medium
ECM: Extracellular matrix
ECL: Enhanced chemiluminescence
FBS: Fetal bovine serum;
GAPDH: Glyceraldehyde-3-phosphate dehydrogenase
iNOS: Inducible nitric oxide synthase
ICA: Icariin
IL-1β: Interleukin-1β
LC3: Microtubule-associated protein3;
MMPs: Matrix metalloproteinases;
mRNA: Messenger RNA
mTOR: Mammalian target of rapamycin
OA: Osteoarthritis
PI3K: Phosphatidylinositol 3-kinase
p62: Sequestosome1,p62/SQSTM1
shRNA: Short hairpin RNA
SW1353: Human chondrosarcoma cell
ULK1: Unc-51-like kinase 1
TNF-α: Tumor necrosis factor-α
